# Shear flow over flexible three-dimensional patches in a surface

**DOI:** 10.1098/rsta.2017.0348

**Published:** 2018-08-20

**Authors:** F. T. Smith

**Affiliations:** Department of Mathematics, University College London, Gower Street, London WC1E 6BT, UK

**Keywords:** floes, flexibility, shear

## Abstract

Slowly varying shear flow is considered over one or more flexible three-dimensional patches in a surface inside a boundary layer. At certain shear values, resonances emerge in which the effects on flow and patch shape are enlarged by an order of magnitude. Fast evolution then occurs: this leads to fully nonlinear unsteady interaction, after some delay, combining with finite-time break-ups to form a distinct path into transition.

This article is part of the theme issue ‘Modelling of sea-ice phenomena’.

## 1. Introduction

This contribution on modelling the interaction between fluid flow and a surface is in the particular context of one or more flexible three-dimensional patches in an otherwise solid surface within a boundary layer or vorticity region astride that surface. The model is perhaps simplistic for a real sea-ice floe or patch in flow but we have in mind nevertheless that the boundary layer can be mainly in the atmospheric flow over a patch or in the sea flow below a patch, depending on how the motion is started up. The patch may represent an ice floe; ice-buckling is also in mind here when the patch shape becomes especially distorted [1]. In terms of air flow over a patch, the present working is on the smaller-scale patches of ice, of length shorter than or not much greater than the representative thickness of an atmospheric boundary layer. Slightly longer patches involve interaction with the flow outside the incident boundary layer and even longer ones are dominated by inviscid effects provided no separation takes place. The boundary layer thickness varies with wind speed but may be typically in the range of tens to hundreds of metres. In terms of water current under an ice patch, similar comments apply restricting the characteristic length scale of the patch.

The incident water boundary layer in this case might be expected to also have a thickness of order tens to hundreds of metres based on the square root of the ratio of the kinematic viscosities for air and water being not especially large and allowing representative current speeds not dissimilar to those of the atmosphere. We note that the issue of whether water (sea) flow or atmospheric (air) flow dominates locally depends to a large extent on how the present background motion originated, whether from a sea surge or from a wind surge, for example.

Related physical interactions concern dynamic fluid–body effects due to ice shards and lumps in the atmosphere and broken-off pieces of ice floe passing under an ice-breaking ship's hull. Related theoretical papers are in [2–4]. The present paper focuses on the influences of fluid-flow vorticity, wall shear, fluid viscosity and three-dimensional features within the sea-ice mechanics area of the current theme issue, along with consideration of the many inherent scales. The intention is to complement direct simulations in order to shed light on three-dimensional mechanics, which enhances upstream and downstream influence of a patch, and to include linear and nonlinear effects, viscous–inviscid interaction and the implications for flow and surface-shape transition. The setting of storms and complex environment is also a factor here.

Section 2 describes the main configuration in non-dimensional terms for laminar unsteady three-dimensional motion of incompressible fluid at high flow rates. Numerical method and results are considered in §3 followed by analysis of modal aspects in §4. Section 5 addresses a resonance which allows nonlinear unsteady effects to play a decisive part. Final comments are presented in §6. The evolution discussed is initially slow over a flexible patch of small slope but ever faster evolution emerges later as the slope increases markedly, leading to transition.

## 2. Shear flow over a hydroelastic surface patch

**Figure 1.**
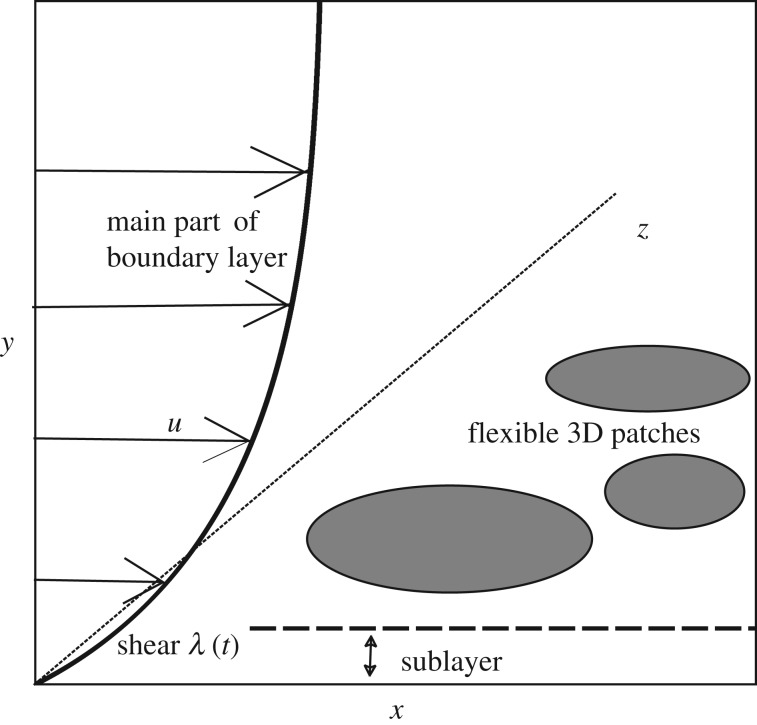
An incident two-dimensional boundary layer flowing in the *x*-direction encounters one or more flexible three-dimensional patches lying in the *x*–*z* plane. The incident near-surface shear flow enters a three-dimensional sublayer that arises in the lower reaches of the surrounding boundary layer.

The three-dimensional configuration has a finite patch or patches of flexible surface which are housed in an otherwise fixed solid surface in the plane *y* = 0 (figure 1). An incident flow of fluid across the configuration has a given uniform wall shear proportional to an *O*(1) factor λ(*t*) which is taken to be quasi-steady, varying over a slow time scale *t*. The flow response and the surface-shape response are coupled and relatively local, with the incident shear being viewed as representing the oncoming motion in the lower reaches (the sublayer) of a surrounding boundary layer, for example, an atmospheric or sea boundary layer depending on the particular context.

In the local properties, our concern is with the so-called condensed flow [1] in which the displacement effects on the rest of the flow field are negligible. Condensed flow occurs in a sublayer near the surface for short length scales streamwise, and corresponding short patches, where the pressures produced are insufficient to significantly displace the surrounding flow outside the sublayer. Mechanically the sublayer response is dominant and the surrounding flow responds only passively. (Longer patches are governed by triple-deck theory involving interaction with the flow outside the incident boundary layer or by inviscid theory provided no significant separation takes place; the latter theory connects with potential flow studies [5–10].) The fluid velocity (*u*, *v*, *w*) in the Cartesian coordinate system (*x*, *y*, *z*) and the induced pressure difference *p* used here are non-dimensional and scaled with respect to a representative length of patch in the streamwise and spanwise directions, a representative height H* in the normal direction, a typical incident shear value S*, and the fluid density and kinematic viscosity ν*. The characteristic slope of the patch is taken to be comparable with 1/*R*_2_ where *R*_2_ is the Reynolds number S*H^*2^/ν* which is assumed to be large, and the sublayer thickness is H*. We note that in the sublayer in the lower depths of a boundary layer these scalings are essentially those of [1]. The governing equations become
2.1*a*
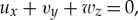

2.1*b*
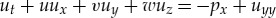

2.1*c*
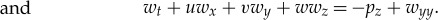


The unsteady contributions in (2.1b,*c*) are included to emphasize the point that there is time-dependence but it begins as slowly varying, with *u*_*t*_, *w*_*t*_ negligible, while the main boundary conditions are
2.1*d*
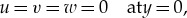

2.1*e*
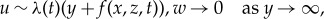

2.1*f*
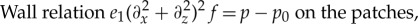

2.1*g*
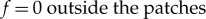

2.1*h*
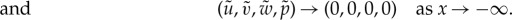


The slow dependence on time is present through the scaled incident shear factor λ(*t*) which is of order unity. The nonlinear three-dimensional interactive boundary layer equations (2.1a,*c*) apply at leading order with the unknown scaled surface shape *f* of the patch being linked to the scaled pressure *p* by means of (2.1f), whereas the wall is assumed to be flat where there is no patch as in (2.1g). To emphasize, the interaction between the flow and the surface shape is two-way in the sense that you cannot determine one without the other. The parameter *e*_1_ is proportional to the flexural rigidity of the patch, it is taken to be a constant of order unity and it is usually negative [1,11]: see remarks on the patch properties at the end of this section. Moreover, the scaled base pressure *p*_0_ relative to the incident pressure level (the latter is taken as zero) could depend on *z* spanwise. A Prandtl transposition 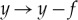 has been applied such that the shape effect *f*(*x*, *z*, *t*) appears in the outer condition (2.1e), leaving the no-slip condition as (2.1d) at the wall. The pressure response *p*(*x*, *z*, *t*) which acts in the streamwise and spanwise balances of momentum in (2.1b,*c*) is also unknown and independent of *y* by virtue of the normal momentum balance which is dominated by *p*_*y*_ having to be zero. The final requirement (2.1h) corresponds to the incident flow condition. The quasi-steadiness is again noted; compare unsteady properties discussed in §5.

The typical patch is assumed to have only small slopes at first and the incident wall shear λ(*t*) to vary only slowly. That suggests we start the analysis with small quasi-steady disturbances, indicating that solutions should be sought in the form 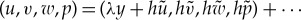 together with a given base pressure level 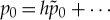 while 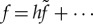 denotes the unknown patch height. Here the scaled height parameter *h* is small. This leads from (2.1) to the linearized system
2.2*a*
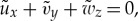

2.2*b*
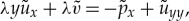

2.2*c*
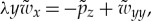

2.2*d*
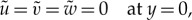

2.2*e*
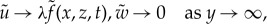

2.2*f*
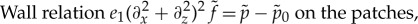

2.2*g*
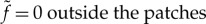

2.2*h*
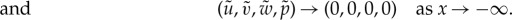
The boundary conditions at the prescribed edge of each patch take the form
2.3
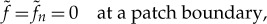
where *n* denotes differentiation in the direction normal to the patch boundary in the *x*–*z* plane. The paper now addresses the linear system (2.2) and (2.3) (before nonlinear effects come to the fore as described in §5).

An array of patches periodic in *z* is supposed of scaled spanwise distance *O*(*b*) between the middle of each patch. If *b* is large, then the solution near each patch is expected to become that of a quasi-isolated patch. On the other hand, an increased spanwise length of patch may produce quasi-two-dimensional behaviour whereas a decrease in that length accentuates the three-dimensional nature of the configuration. Concerning the wall relation (2.1f) and hence (2.2f) it contains no unsteady terms proportional to *f*_*t*_ and *f*_*tt*_ since the mass density and the damping constant are supposed comparatively small and likewise contributions from lower derivatives such as (*δ*^2^_*x*_ + *δ*^2^_*z*_)*f* and *f* itself are neglected on the grounds of negligible longitudinal tension and spring stiffness in effect.

## 3. Numerical method and results

### (a) Methodology

Extra ellipticity is present because of the three-dimensional effects [12], in the form of significant upstream influence; in the two-dimensional case the upstream influence ahead of the *x*-station of the first patch is insignificant at leading order. So strictly extra boundary conditions are required but these become clear as we proceed.

We proceed by successively iterating between the flow and shape problems. First, the skewed shear method [12] is used for the flow. The momentum balances are combined by adding the *x*-derivative of (2.2b) to the *z*-derivative of (2.2c) which, taking (2.2a) also into account and using the quasi-shears defined by 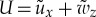 and 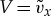, yields the formulation
3.1*a*
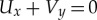
and
3.1*b*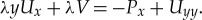 (The unknown response *P* has a Laplacian form; see (3.3a).) This is together with the conditions
3.1*c*
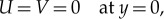

3.1*d*
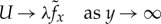

3.1*e*
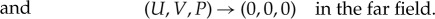
Here the individual patch shape 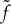 is treated as known at this stage of iteration whereas the quasi-pressure *P* is a response to be found. Further, the formulation above is that of a two-dimensional flow problem in which *z* acts only as a parameter. The solution at each *z* can be obtained by a transform approach and gives the quasi-pressure result [1] explicitly as
3.2*a*
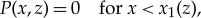

3.2*b*
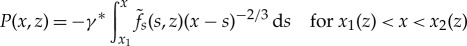

3.2*c*
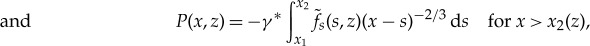
with the (so far) passive *t*-dependence suppressed. Here the constant γ* = − 3*Ai*^′^(0)λ^5/3^/*Γ*(1/3) is positive, approximately 0.289838λ^5/3^, and *x*_1_, *x*_2_ denote the *x*-values of the front and rear of the patch, respectively, at a fixed *z*; if the fixed *z*-plane does not intersect any patch then (3.2a) applies throughout. Hence *P*(*x*, *z*) can be determined directly everywhere in the *x*–*z* plane. On the other hand, the manipulation leading to (3.1b) shows that
3.3*a*
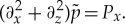
So the pressure 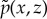 is then to be found by solving the forced Laplace equation (3.3a) for 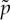, for example, by an iteration method, with the right-hand side defined by derivatives of (3.2a,*c*) and with the requirement that
3.3*b*
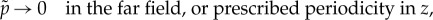
depending on the context.

Second, a relaxation method is used for the shape problem which consists of (2.2f), (2.2g), (2.3) with 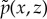 assumed given by the latest update as in the previous paragraph and with a specified constant 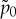 value. The field equation is treated as
3.4
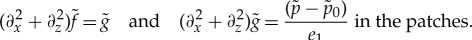
These two equations are solved iteratively for 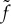, 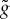 subject to second-order accurate representations of the edge conditions (2.3) and subject to relaxation. The shape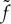 thus obtained is fed back into the flow problem of (3.2) and (3.3) to deduce a new pressure, which is fed again into the shape problem, and so on until the overall iterations converge. The above reasoning is presented mostly for a single patch but similar considerations apply for multiple patches and periodic configurations. Note Fourier transform methods might be considered but these tend to be hindered in their practicability by the patches being of finite length.

### (b) Numerical results

There are many system parameters. We chose certain specific parameter values to illustrate the major points of interest here, for a spanwise periodic array of rectangular patches. The base pressure level 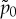 was set as unity without loss of generality. The computations kept the spanwise period *b* equal to 5, the streamwise length of the patch fixed at 2, the coefficient *e*_1_ = − 1 and the incident shear λ as unity (compare §4 below). We varied the spanwise length *l* of the patch and also the properties of the numerical grid.

**Figure 2.**
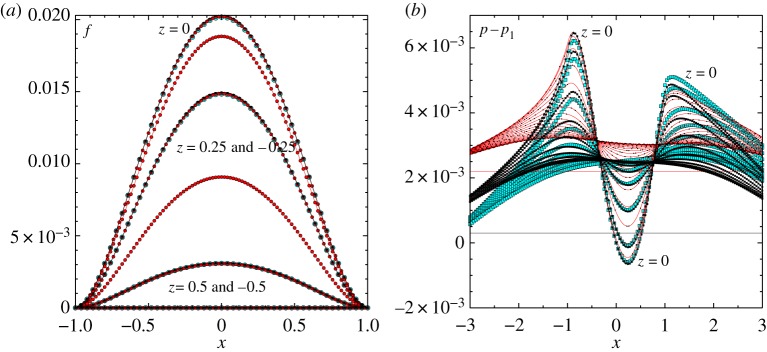
Solutions with a patch of scaled length 2, span 1.25, base pressure *p*_0_ = 1 and incident shear λ = 1. Effects of *x*, *z* grid sizes (201, 201 points; 401, 401; 401, 201) for end values (−4, 4), (−4, 4), (−8, 8) in *x*, respectively, are also included. (*a*) Scaled shape *f*. (*b*) Scaled pressure difference *p* − *p*_1_, where *p*_1_ is the pressure at the origin; horizontal lines indicate the values obtained at the ends of the grid; *z* = − 2.5(0.125)2.5.

**Figure 3.**
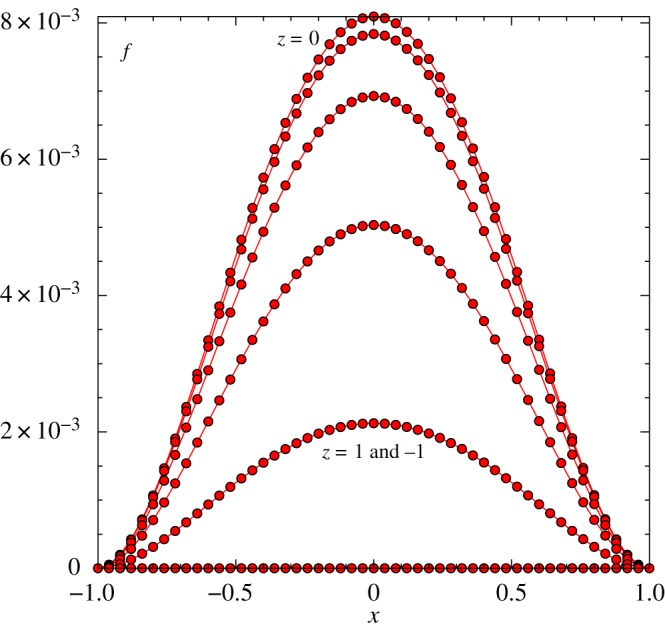
As in figure 2*a* but with a span of 2.5. Grid has 201 points in *x*, *z* with *z*-step of 0.025.

Results are shown in figures 2 and 3. The interactive shapes 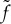 and pressure difference distributions 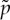 are given in figure 2 for the case of a spanwise extent *l* = 1.25. The 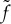 solutions are similar to those in the two-dimensional scenario [1] but now vary with *z*, with a maximum occurring along the centreline and only small values arising near the spanwise edges. The pressure difference solutions 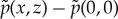 at each *z*-value are likewise reminiscent of the two-dimensional results but, in the spanwise direction, are maximal at the centreline and they display the spread of pressure outside the patch, to the sides as well as ahead and behind the patch. Grid tests given in figure 2 show 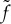 agreeing well from grid to grid throughout, whereas the 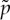 solutions are shifted up or down while maintaining their basic shape. Figure 3 is for the same parameter values except that the spanwise extent *l* is doubled to 2.50. Here 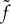, 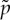 in particular are both increased significantly in magnitude.

Upstream influence appears in all cases as anticipated earlier. There is in addition a considerable wake effect downstream. The latter appears to affect the level of the pressure solutions throughout. These findings indicate the potential value of conducting a mode analysis for the sake of understanding the shape and pressure features more.

## 4. Mode analysis and resonance

The following modal approach is found to shed light on the interactions above. The base pressure 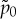 is supposed now to depend on *z* according to cos(*βz*) say where the periodicity coefficient *β* is a prescribed constant. Considering all *z*-dependence to be in the modal form 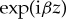 or more generally a summation of such terms, thus
4.1
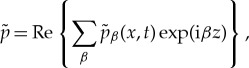
we see that the relations (3b) between *P*, 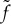 then remain intact with *x*_1_, *x*_2_ being constants. The relation (3.3a) linking 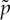, *P* however is transformed to
4.2*a*
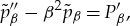
where the prime denotes a derivative with respect to *x*. Also the wall equation (2.2f) or (3.4) becomes
4.2*b*
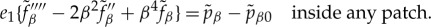
Here 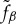, 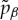, *P*_*β*_ are complex functions of *x* in general and 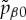 is a given complex constant. The latter could vary from patch to patch in principle. The boundary conditions are
4.3*a*
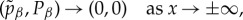

4.3*b*
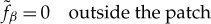

4.3*c*
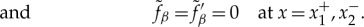
This is currently for a single patch in the streamwise direction; thus (4.3b) applies for *x* < *x*_1_ and *x* > *x*_2_. The task is to solve (4.2), (4.3) together with (3.2).

To solve we address the streamwise ranges ahead of the patch, in the patch and downstream of the patch in turn. Ahead of the patch (4.2a) holds but with *P* identically zero by virtue of (3.2b). So from (4.2a) the pressure is simply
4.4*a*
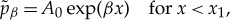
where the constant *A*_0_ is to be determined and condition (4.3a) is satisfied at −∞ as required. In the patch itself the solution can be written in the form
4.4*b*
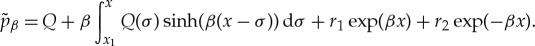
Here *r*_1_, *r*_2_ are unknown constants while *P*_*β*_ is expressed as *dQ*/*dx* with *Q* unknown. Thus (3b) gives the relations
4.4*c*
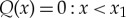

4.4*d*
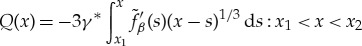

4.4*e*
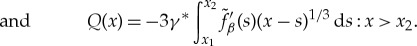


**Figure 4.**
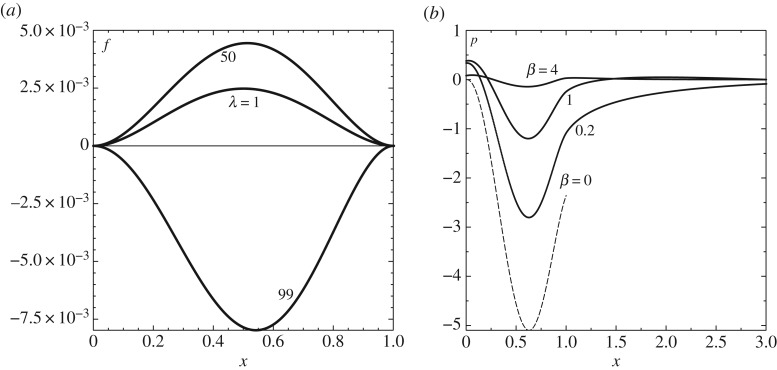
Modal analysis. (*a*) Shape 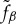 (denoted *f*) for λ = 1, 50, 99 when *β* = 1; resonance occurs at a λ value between 50 and 99 (figure 5). (*b*) Pressure 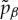 (denoted *p*) for varying *β* with λ = 50.

The flow properties (4.4) are coupled with the wall properties of (4.2b) to determine the scaled wall pressures and shapes numerically, bearing in mind that 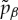, 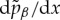 are continuous at the start of the patch and 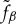, 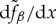 are zero at both ends of the patch. It follows that *r*_1_ = *A*_0_ while *r*_2_ is zero. The iterative method used marches forward in *x*, given a guessed 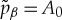 at *x* = *x*^−^_1_, and the march continues to a downstream location *x*_∞_ sufficiently far beyond *x* = *x*_2_ to reveal exponential growth in *x* downstream of the patch at any typical iteration. The value of *A*_0_ is then adjusted by means of Newton iteration to produce zero such growth downstream. Solutions are shown in figure 4.

**Figure 5.**
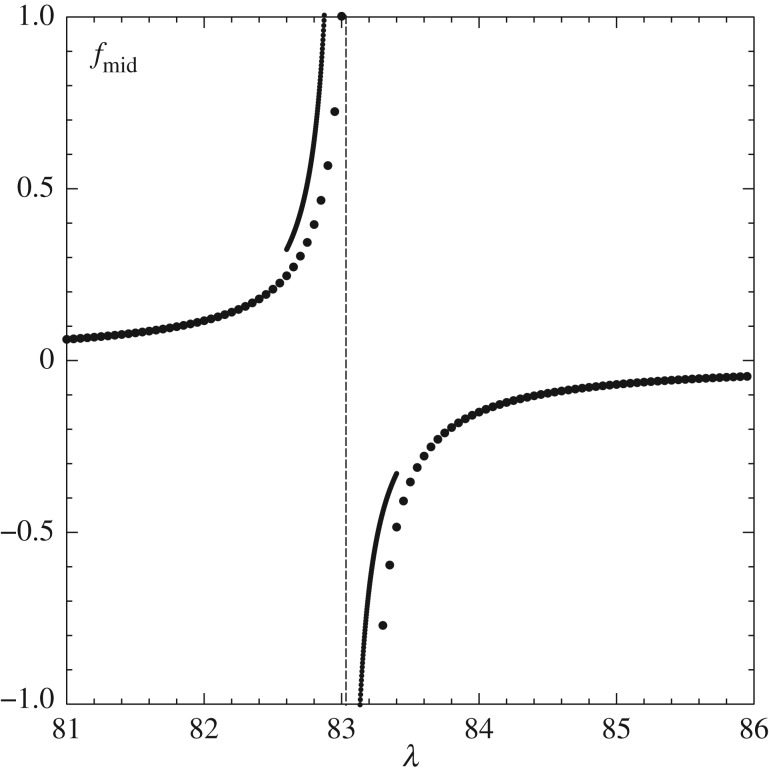
The mid-height *f* (=*f*_*mid*_) at 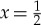 versus λ for fixed *β* = 1. Dotted curves: from medium grid. Solid: finer grid. Dashes: resonant value from finer grid.

Figure 4*a* shows the shape solutions for a fixed *β* of unity with the incident shear λ taking the values 1, 50, 99 and 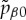 again being unity. Here (*x*_1_, *x*_2_) = (0, 1). More phenomena of interest are found to occur as λ increases. We observe that the typical 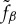 value increases monotonically with λ until a certain critical value between 50 and 99 is reached, after which 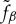 becomes negative: this leads on to the investigation of resonance below. Figure 4*b* gives the pressure results for various *β* values with λ fixed at 50. For higher values of *β*, the pressure variation is relatively slight but it increases monotonically with decreasing *β* and the upstream and downstream influences become more apparent. For *β* values greater than about unity a moderate downstream end *x*_∞_ is sufficient, whereas lower values require a much increased end range, with pronounced pressure responses arising then, and the computation becomes more sensitive. Decreasing *β* eventually leads to increased streamwise extent, decreased amplitude of upstream influence and increased downstream influence. The two-dimensional solution associated with zero *β* is also shown for comparison.

Small *β* values corresponding to relatively large spanwise length scales admit further analytical insight. Bearing in mind that zero *β* produces no upstream influence at this level we find a two-tiered response holds: for *x* of *O*(1) the two-dimensional result with 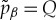 applies, giving 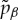 decaying as *x*^−2/3^ downstream, whereas further downstream for larger *x* of *O*(*β*^−1^) the three-dimensional nature reasserts itself. From (4.2a) with *Q*^′′^ on the right-hand side and scaling *x* = *β*^−1^*X* the pressure is
4.5
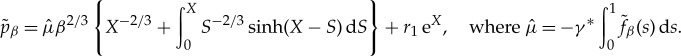


Here 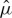 stems from the two-dimensional zero-*β* result, where the dependence on the cross-sectional area of the distorted patch is notable, and (*x*_1_, *x*_2_) = (0, 1) again. The condition of no exponential growth at large positive *X* therefore yields the prediction
4.6
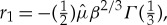
to leading order. A calculation of 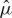 based on the two-dimensional shape 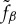 in (4.5) leads to the numerical value *r*_1_ = 0.603 from (4.6) for the case *β* = 0.2. Also this three-dimensional effect determines the upstream influence that occurs over the long *O*(*β*^−1^) length scale in view of (4.4a) with *A*_0_ given by *r*_1_. As an approximation the numerical value just above is not far from the pressure values obtained in the earlier results of figure 4 at the beginning of the patch for a *β* of 0.2. In addition the relative correction of order *β*^2/3^ coupled with the two length scales in *x* helps to account for the sensitivities in computing at small *β* values (and explains pressure sensitivity in figure 2*b*).

Large *β* values similarly allow analysis. Upstream influence lengths are small of order *β*^−1^ here. The main response takes place over the patch where the shape 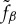 is given approximately by 
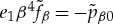
 from (4.2b), since 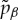 is of order *β*^−6^, except near the ends where there are thin zones in which the *x*-variation is of order *β*^−1^. Results for *β* = 4 in figure 4 tie in with the approximation in terms of the 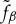 maximum value.

Resonance is found to occur. For a representative fixed *β* of unity, the results of varying the incident wall shear λ are presented in figure 5. These indicate a critical value λ = λ_1_ (about 83.0) at which the shape and pressure response become unbounded on linear grounds, or ‘intensification’ takes place (giving a weakly nonlinear stage followed by a strongly nonlinear one, depending on details, see below). In fact, an infinite sequence of such critical values or eigenvalues λ = λ_*n*_ is obtained. Such resonance is present for any *β* value.

## 5. Resonance leads to nonlinear evolution and transition

**Figure 6.**
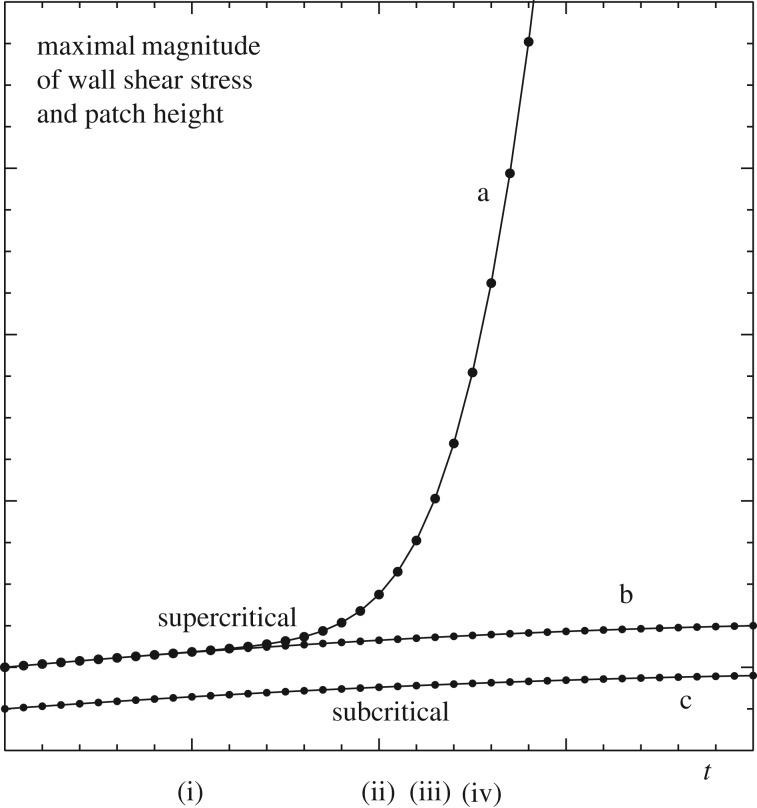
Effects of changing incident shear λ. Curve a shows maximal local wall shear and patch height when λ varies slowly as in b from subcritical to supercritical. When λ variation is as in c the response remains close to c. Time scales (i–iv) are slow, fast, faster, faster and increasingly nonlinear.

The resonance phenomenon has an interpretation in terms of flow transition as depicted in figure 6. Clearly when the wall shear λ(*t*) of the incident flow acquires certain critical values the flexible patch, after some delay due to initially slow variation, rises or falls to a magnitude much greater than is otherwise the case. This intensification along with a nonlinear blowup which is accompanied by a shortening time scale as in [1] points to a turbulent trip effect being produced over a wide parameter range. Both shear and wall flexibility are required for the present intensifications and fast growth mechanisms to occur. The intensification present here leads to a non-standard path into transition from low amplitudes, as figure 6 indicates by showing schematically the process of accelerating effects that yield strongly nonlinear evolution. During that evolution the full governing equations (2.1a–*c*) re-enter play. This is followed by finite-time blowup as in Smith [13] and Peridier *et al.* [14] which provokes the even faster evolution described by Bowles *et al.* [15] (see also [16,17]) with further restructuring and deep transition towards turbulence taking place. The boosted nonlinear behaviour here arises directly from the intensification associated with a critical λ, which represents variation in the surrounding flow conditions.

## 6. Further comments

The study of incident shear effects seems unusual for flow over three-dimensional flexible patches and in the sea-ice context especially. This may be shear in the sea or wind shear in the air, depending on the setting, and there could even be interaction between the sea and the air through kinematic conditions. When the incident shear varies slowly the initial effects with small disturbances allow analytical insight. More significant however are the implications for transition: in brief at critical (resonant) shear values considerable unsteadiness enters first via a weakly nonlinear behaviour at or near resonance and secondly through blowup yielding fully nonlinear behaviour, as in [1]. The distortion of the patch shape also becomes intensified, which may be relevant to the buckling of ice sheets. Other nonlinear physical features also enter in various ways, for instance, through flow separations arising. All of this nonlinearity is alternative to the interesting nonlinearity in [8–10].

Cases of many patches and periodic arrays, which have biomedical application as well, deserve further attention. It would be interesting to examine influences from mass density, damping, longitudinal tension and spring stiffness [1,11], while there is a clear need for more three-dimensional interaction studies to admit realistic shapes and shorter time scales. Models of storm effects are also of interest here. One such is concerned with wall-bounded shear flow containing freely moving bodies, giving dynamic fluid–body interactions [2–4], where nonlinear, many-body and flow-separation effects have still to be incorporated.

## Data accessibility

This article does not contain any additional data.
